# Proceedings of Veterinary Societies

**Published:** 1888-07

**Authors:** Wm. Herbert Lowe

**Affiliations:** Secretary


### PROCEEDINGS VETERINARY SOCIETIES.

I.—UNIVERSITY OF PENNSYLVANIA.—The one hundred and thirty-second annual commencement of the University, and the second of its Veterinary Department, was held on Wednesday, June 6, 1888, at the Academy of Music, in Philadelphia. The Degree of *Veterinariæ Medicinæ Doctor* was conferred upon the following gentlemen:

Frank B. Bachman, Strasburg; Helmuth C. Balzer, Meriden, Conn.; Leo Breisacher, Detroit, Mich.; Howard B. Felton, Olney; Robert Formad, Philadelphia; Caspar Garrett, Lansdowne; Guldin R. Hartman, Philadelphia; Louis Olry Lusson, Philadelphia; Antoni Maurise, Philadelphia; Leon N. Reefer, Meadville; William Hodgson Ridge, Trevose, Pa.; Albert F. Schreiber, Philadelphia; John Z. Tintsman, Philadelphia; William B. Werntz, Philadelphia.

*Honorable Mention:*—For the best Thesis, Howard B. Felton; for General Excellence, Leon N. Reefer.

The fourteen gentlemen who successfully terminated all of their studies represent a matriculation roll of twenty-eight on their entrance to the school in 1884. Of these, eight failed in their studies the first year, eight more failed in the second year; the remaining fourteen were augmented in the third to sixteen by the addition of two from the preceding class.

II.—MONTREAL VETERINARY COLLEGE.—At the twenty-second session of the Montreal Veterinary College the chair was occupied by Mr. S. N. Blackwood, J. P., member of the Board of Agriculture, and amongst others present were G. Leclerc, secretary to the Board of Agriculture, J. W. Gadsden, M.R.C.V.S., Philadelphia; W. Bryden, V.S., Boston; J. Crawford, J. Kimball, and many of the relatives of the students.

The Chairman, on rising to present the prizes said that the examinations this year showed that the students possessed a better knowledge of their profession than in previous years, and hoped those who were leaving the college would continue to uphold the honor of their Alma Mater. He had taken a good deal of interest to find out how the past graduates had progressed, and has discovered that fourteen of them averaged an annual income of $6000 a year. There was no reason, seeing that this country was yet in its infancy, that those present, if they only put their heart into the branch of study they had choser, should not attain even to greater results.

Dr. J. W. Gadsden, of Philadelphia, and Mr. W. Bryden, V.S., Boston, also spoke a few words of encouragement to the students.

Dr. Mills hoped that they would not forget their Alma Mater. It was due to the college itself that, no matter in what part of the world they located, they should correspond with them. It gave a stimulus to future students to try and be as successful as those who had gone before. He wished them long life and prosperity.

The Chairman then distributed the prizes as follows:

*Best General Examination*—Silver medal, the gift of the Council of Agriculture, to the student who obtains the highest percentage of marks on all the subjects for his two last years of study, awarded to H. R. Macaulay.

*Practice of Medicine and Surgery* (senior class)—1st prize, George C. Becket; 2d prize, James B. Paige; honorable mention, H. R. Macaulay.

*Veterinary Anatomy*—1st prize, H. R. Macaulay; 2d prize, J. B. Paige; honorable mention, John Robertson.

*Cattle Pathology*—1st prize, J. B. Paige; 2d prize, H. R. Macaulay; honorable mention, — McGarth.

*Chemistry*—1st prize, Robert Darling; 2d prize, J. H. Roberts.

*Physiology*—1st prize, H. R. Macaulay; 2d prize, J. M. Parker.

*Materia Medica*—1st prize, G. A. Wieland; 2d prize, J. G. Harris.

*Pathological Anatomy* (presented by Dr. Johnston for proficiency in microscopic work and autopsy)—H. R. Macaulay.

JUNIOR CLASS. *Practice of Medicine and Surgerg*—1st prize, J. G. Harris.

The Chairman then called upon Principal McEachran to present the diplomas of the association. The Principal, in a short but pithy speech, exhorted them not to lay aside studies after they had left the college, but to continue them, and try and raise themselves to as high a standard as they could possibly reach, and they would find that their efforts would be crowned with success. He then presented the following students, who had fulfilled the requirements of the curriculum and obtained the necessary percentage of marks in their written examinations, presented themselves and successfully passed, viz.:—Messrs. Becket, Craig, Dawes, Macaulay, McGarth, Miller, Munro, Murphy, Paige, Roberts, Robertson and Smith.

A vote of thanks was then passed to the Chairman, after which the session closed.

III.—ONTARIO VETERINARY COLLEGE.—The examinations for the graduating class of 1888 took place in the museum of the College. The Board of Examiners met on Monday, the 26th March, and the work was concluded on the 29th. During those four days a large number of students were examined. The majority of the applicants acquitted themselves well, the percentage of failures being small. Considering the strict and searching nature of the examinations and the care bestowed upon the consideration of each case by the Board, the students themselves, as well as the teaching staff of the college, are to be congratulated.

One hundred and twenty-five gentlemen graduated upon this occasion.

The closing exercises of the college took place in Richmond Hall, on the 29th. Several ladies graced the occasion by their presence.

Professor Smith, F.R.C.V.S., Principal of the College, occupied the chair, and opened the proceedings by calling for the reading of the list of graduates with the prize and honor list.

The following gentlemen among others presented the medals and prizes: Dr. Daniel Wilson, Dr. Thorturn, Dr. Duncun, Dr. Cowen, Dr. Peters, Rev. Dr. Wild, Ald. Frankland, Dr. Cowan, Dr. Coleman. In the presentation of the grand gold medal for the student passing the best general examination, the fact was noted that the competition was very keen, the successful competitor being W. Payson Schwin of Indiana, U. S. The announcement of Mr. Schwin's success was greeted with warm applause by the graduates and others who filled the hall.

THE GRADUATING CLASS.

Ackland, Geo. F., Forfar.
Allis, N. H., Nyalusing, Pa.
Anderson, J. P., Guelph.
Anderson, Robert, Combor.
Armstrong, James A , Stratford.
Austin, David G , Essex Centre.
Bade, Thomas, Caledon.
Baker, Trayton F. F., Oakville.
Baker, Wallace L., Lafayette, N. Y.
Balliett, A. H., Ballietsville, Pa.
Bell, Charles F., Arcanium, Ohio.
Berry, Robt. G., Sherbrooke, P. Q.
Bland, Charles, Lincoln, England.
Bland, J. W., Calgary, N. W. T.
Bowker, Glenn, Groton, N. Y.
Bracken, W. J., Brampton.
Booker, Frank, Carlingville, Ill.
Bradley, J. E., Gananoque.
Buckingham, Jabez D., Essex Centre.
Burger, Wm., Hornby.
Burke, J. C., St. Thomas.
Brackin, G. E , Caledon E.
Campbell, Arthur E., Buffalo, N. Y.
Campbell, James, Strathroy.
Campbell, W. D., Ridgetown.
Carpenter, Howard T., Manhattanville, Ks.
Carpenter, Tom, San Francisco, Cal.
Carter, Geo. H., Guelph.
Chase, Jesse M., Sherwood, N. Y.
Clute, H. P., West Shelby, N. Y.
Collins. George, Hespeler.
Cook, F W. Hutchinson, Ks.
Cornell, Chester E., Thedford.
Coutts, J. J., Crosshill.
Coxe, William, Nassagawayn.
Dengler, H O., Quakertown, Pa.
Dann, William, Lucan.
Davies, Charles, Alton.
Edwards. Edward, Lima, Ohio.
Erb, N. S., Guelph.
Findlay, James J., Williamstown.
Fox, John H., Guelph.
Gable, E E. Meadville, Penn.
George, J. H., Crampton.
Gilbank, F. G., Cirr.
Glass, Wm. H., Australia.
Graham, Joseph, Port Perry.
Graham, Newton, Zephyr.
Gruber, C. D., Obolds, Pa.
Green, David, Ridgetown.
Hagyard, E. W., Lexington, Ky.
Hambidge, Thomas, Mount Hope.
Heighway, J. G., London.
Hickingbottom, Robert, Balsam.
Hill, James A. T.. Chicago, Ill.
Hoffman, Frank W., Waterloo. Ind.
Inger, J. D., Strawberry Point, Iowa.
James, A., O tawa.
Jackson, A. W., Abingdon.
Johnson, James H., Croton.
Johnson, S. H., Chesley.
Johnson, Benjamin, Davenport.
Johnstone, J. A., Trafalgar.
Kennedy, John T., Bloomington.
Kent, F. B., Bracebridge.
Kimmell. D. E., Greensburg, Pa.
Kydd, W. H., Warren, Ill.
Lake, Constant, Wooster, Ohio.
Lambertus, Joseph, Teeswater.
Lawson. John A., Walkerton.
Leary, Daniel, Kendall, N. Y.
Lindsay, W. H., Hornby.
McCapes, Adelbert, Vermilion, Da.
McGregor, J. D., London.
McKenna, W. C. S., Brampton.
McLean, W. A., Lucan.
McQueen, E. D., Port Dover.
McMahon, D. T., Chicago, Ill.
Macdonald, Fred, S., Toronto.
Merillat, L. A., Kochs, Ohio.
Millar, John James, Colgary, N. W. T.
Morris, Claude D., North Reading. N. Y.
Moulter, G. H., Fayetteville, N. Y.
Newby, Thos. B., Charleston, Ill.

Orr, A. E., Milford, Kansas.
Paget, Henry A., Loughborough, England.
Paige, H. E., Amherst, Mass.
Paxton, John S., Fredericksburg, Ohio.
Phillips, John R., Dewittville, N. Y.
Porter, Joseph W., Mount Vernon.
Powell, J. H., Thedford.
Power, Richard H., Barrie.
Quin, Abner H., Edmonton.
Readhead, R. T., Lowville.
Robinson, Samuel G., Galt.
Roe, J. S., Milverton.
Roe, J. A., Milverton.
Rossiter, A. [illegible], Salem, Oregon.
Rossiter, Edwin W., Salem, O.
Rowe, H. M., Strathroy.
Rowlin, G. H., Hamilton.
Russell, W. T., Nashville, N. H.
Schwin, Payson E., Middleburg, Ind.
Scott, Walter J., Duncrieff.
Scott, Andrew D., Duncrieff.
Schaffter, E. P., Mount Eaton, Ohio.
Shepard, Edgar H., Geneva, Ohio.
Sherrich, F. W., Tarrs, Pa.
Sihler, Charles J., Simcoe.
Smith, R. V., St. Mary's.
Smythe, T. H.: Grand Forks, Dak.
Speirs, Henry J., Manitoba.
Stevens, David F., Mount Morris, Ill.
Sutherland, Geo. H., East Saginaw, Mich.
Sloan, J. Langtry, Churchill.
Thompson, James D., Sharkleyville, Pa.
Thompson, Lewis H., Strathroy.
Vanantwerp, E. A., Grand Rapids, Mich.
Walker, Joseph J., Londesborough.
Walkington, John J., King.
White, James S., Elmira.
Wicks, Arthur G., Springfield, Mass.
Williams, George C., Fingal.
Wilson, John E., Thornton.
Zug, Frank M., Clarence, N. Y.

After the presentation of the prizes, Professor Daniel Wilson, of Toronto University, was called upon, and in response expressed the pleasure he experienced at being present. He spoke eulogistically of the President of the Ontario Veterinary College, Professor Smith, referring to the wonderful success which the college, through his energy and ability, had achieved. Students flocked to it from all parts of the continent, and, considering the solid advantages which they secured by attending here, their presence in such numbers was not surprising. He made special and pleasing allusion to the warm reception given to the winner of the gold medal, showing, in his opinion, two gratifying facts: first, that there was no petty jealousy among the students of the college; and, second, that they had perfect confidence in the fairness of the examining board.

Rev. Dr. Wild spoke of the college and its work in reference to the prevention of cruelty to animals. He considered the work done here would go farther in the way of prevention of cruelty than even the honored society which takes up this work as its special object. After a graceful reference to the presence of the ladies, and a humorous one to the first occasion on which he had met the students, the Doctor concluded by emphasizing his opinion of the importance of the study of comparative pathology, which was so much facilitated by the course of teaching in the Ontario Veterinary College.

Alderman Frankland was received with much enthusiasm, as was fitting, for he has proved himself for years to be the warm friend of the college, the students and the graduates. As one of the largest cattle exporters of the continent, he has a special interest in all veterinary matters. He spoke words of encouragement and congratulation to the large graduating class, pointing out that the members must emulate the progress of former graduates, many of whom had attained to high positions.

Alderman Dodds spoke of the long friendship which had existed between himself and the President of the college. He eloquently urged the graduates to keep in view the energy of him who had made this college what it is.

At the conclusion of Alderman Dodds' address, L. Carpenter, V.S., of U. S., stepped upon the platform, and, on behalf of the members of the graduating class, asked Professor Smith to accept a magnificent photograph of themselves as a token of their esteem for him. He read an excellent address, expressive of their attachment to the college, and the various teachers in connection therewith. Professor Smith, in feeling terms, accepted the gift, remarking upon the pleasure which such manifestations of kindness caused, and upon the fact that the relations between teachers and taught had always been of the most cordial kind in the Ontario Veterinary College. Excellent speeches of Dr. W. Cowan, V.S., of Galt, and Dr. A. O. F. Coleman, V.S., of Ottawa, former graduates of the college, closed the proceedings.

IV. New Jersey State Veterinary Society.—The last regular meeting of this society was held on Thursday, April 26, 1888, at Taylor's Hotel, Jersey City, with Dr. J. C. Corlies, President, in the chair, and a large attendance of members from various parts of the State.

The minutes of the Newark meeting were read and adopted.

Dr. J. Serling (graduate of a German college), a practitioner of Hoboken, was elected to membership after considerable discussion. It was held by some of the members present, that although Dr. Serling was a practitioner of Hoboken, yet he resided in New York, and, not being a resident, was not entitled to membership in the Association.

Dr. Vreeland, of Jersey City, a recent graduate of the A. V. C., was proposed for membership.

The subject of contagious pleuro-pneumonia in Hudson County and elsewhere in the State received considerable attention.

It was the sense of the meeting that Dr. Dimond and his deputies were doing good work in Hudson County and deserved the hearty support of not only every veterinarian in the county and State, but of the stockowners and the public generally. Dr. Nayler thought there was too much politics in the matter, and others expressed the fear that if politics were allowed to get a hold, the usefulness of the Board would be greatly impaired.

Much time was taken up with the revision of the Constitution and By-Laws of the Association. The fact that the Association is now so incorporated, under the Act of the Legislature for the promotion of veterinary science and art, that only graduates from chartered veterinary colleges are eligible to membership, simplifies the by-laws greatly.

Article II. of the Constitution was amended to read that the "New Jersey State Veterinary Society has for its object the promotion of fraternal feeling among its members, the welfare of the veterinary profession, in general, and of New Jersey in particular, aiming to protect the rights and privileges of practitioners, and to elevate the standard of the profession by scientific intercourse."

Hereafter, instead of tri-annual meetings, the Association will hold quarterly meetings in various parts of the State, and a banquet will be held each year at the annual meeting, which occurs in the month of August.

The secretary was instructed to have the new by-laws printed The Association adjourned to meet at Long Branch, August 4th, when the annual election of officers will be held.

WM. HERBERT LOWE, D.V.S., Secretary.

